# High Immune Response Rate to the Fourth Boost of the BNT162b2 Vaccine against the Omicron Variants of Concern among Liver Transplant Recipients

**DOI:** 10.3390/v14122769

**Published:** 2022-12-12

**Authors:** Yana Davidov, Victoria Indenbaum, Nofar Atari, Limor Kliker, Keren Tsaraf, Keren Asraf, Oranit Cohen-Ezra, Mariya Likhter, Orna Mor, Ram Doolman, Yael Weiss-Ottolenghi, Tammy Hod, Arnon Afek, Yitshak Kreiss, Yaniv Lustig, Gili Regev-Yochay, Michal Mandelboim, Ziv Ben-Ari

**Affiliations:** 1 Liver Diseases Center, Sheba Medical Center, Tel Aviv 52621, Israel; 2 Central Virology Laboratory, Ministry of Health, Tel HaShomer 52621, Israel; 3 Sackler Faculty of Medicine, Tel Aviv University, Tel Aviv 69978, Israel; 4 The Dworman Automated Mega Laboratory, Sheba Medical Center, Tel HaShomer 52621, Israel; 5 Infection Prevention & Control Unit, Sheba Medical Center, Tel HaShomer 52621, Israel; 6 Renal Transplant Center and Nephrology Department, Sheba Medical Center, Tel HaShomer 52621, Israel; 7 General Management, Sheba Medical Center, Tel Aviv 52621, Israel

**Keywords:** BNT162b2 mRNA, fourth dose, liver transplant recipients, breakthrough infection, immune response, side effects BNT162b2 mRNA vaccine

## Abstract

The immune response of liver transplant (LT) recipients to a third dose of the BNT162b2 mRNA vaccine significantly waned after four months. We aimed to evaluate the immune response and breakthrough infection rates of a fourth dose against the Omicron variants among LT recipients. LT recipients who had no past or active SARS-CoV-2 infection and received three doses of the BNT162b2mRNA vaccine were included. Of the 73 LT recipients, 50 (68.5%) received a fourth dose. The fourth dose was associated with a significantly higher positive immune response than the third dose. Receptor-binding domain (RBD) IgG and Omicron BA.1 and BA.2 neutralizing antibodies were determined at a median of 132 and 29 days after the third and fourth vaccines. They were 345 binding antibody units per milliliter (BAU/mL) vs. 2118 BAU/mL (*p* < 0.0001), 10 vs. 87 (*p* < 0.0001), and 15 vs. 149 (*p* = 0.001), respectively. Breakthrough infections were documented among nine (18%) LT recipients after the fourth dose and among seven (30.4%) patients following the third dose (*p* = 0.2); 93.5% of breakthrough infections were mild. The infection rate after the fourth dose was higher among diabetic vs. nondiabetic recipients (33.3% vs. 6.9%, respectively; *p* = 0.02). Further studies are needed to evaluate additional factors influencing the breakthrough infection rate among LT recipients.

## 1. Introduction

When compared to healthy subjects, the immune response to the BNT162b2 mRNA vaccine against SARS-CoV-2 among solid organ transplant (SOT) recipients was significantly impaired [1,2,3,4,5,6]. After the second vaccine dose, patients showed an initial response, which waned significantly 5–6 months later, but was restored by the third vaccine dose [5,6]. In liver transplant (LT) recipients, the immune response to the third dose was significantly enhanced compared to that of the second dose, increasing from 68% one month after the second dose to 98% three weeks after the third [6]. As expected, after the third dose, the immune response waned more profoundly among LT recipients than among immunocompetent individuals (manuscript submitted). Earlier studies, including ours, also found that the type of immunosuppression therapy impacted the immune response of LT recipients to the second and third vaccine doses [1,6] (manuscript submitted).

The recent emergence of the SARS-CoV-2 Delta and Omicron variants of concern (VOC) threatens the effectiveness of the currently available vaccines [7,8,9]. In late December 2021, with the emergence of the Omicron BA.1 (B.1.1.529) variant, the prevalence of confirmed infections rose sharply in Israel [7]. The Omicron variants have a higher effective reproduction number compared to the Delta variant [10]. However, they are less severe. In South Africa, Maslo et al. reported a lower rate of admission during the predominance of Omicron compared with the rate of hospitalization during the predominance of Delta, which was 41.3% and 69%, respectively [11]. Additionally, they reported a significantly lower rate of intensive care admission and death (29.9% vs. 18.5% and 29.1% vs. 2.7%, respectively) [11]. Administration of a fourth dose of the vaccine (either mRNA-1273 or BNT162b2) increased neutralizing antibody titers among healthcare workers (HCWs) to levels similar to those measured after the third dose [9]. Among young HCWs, the fourth vaccine dose prevented symptomatic disease but showed little efficacy against infections [9]. In the older population (≥60 years), the rates of confirmed COVID-19 infection and severe disease after four doses was lower by two-fold and three-fold, respectively, as compared to after the third vaccine [7].

Since March 2022, new Omicron variants emerged and BA.2 (B.1.1.529.2) became dominant in many countries, including Israel [12]. The BA.1 and BA.2 variants share multiple common mutations, but each has unique mutations [12,13]. Several studies reported reduced neutralization efficiency after infection or vaccine-induced immunity to Omicron among healthy and immunocompromised individuals [9,12,14]. The Omicron variant displayed up to 58-fold reduced neutralization activity in convalescent plasma or sera and 10-fold reduced neutralization activity after mRNA vaccines [9,12].

Data on the effectiveness of the fourth vaccine dose among SOT patients are limited. Peled et al. reported an improved immune response among heart transplant (HT) recipients after the fourth BNT162b2 vaccine dose (positive immune response improved from 61.4% to 80.7% before and after the fourth dose, respectively) [15]. The fourth dose also improved the immune response among kidney transplant (KT) recipients. Midtvedt et al. reported a 42% positive immune response among patients who failed to respond to the third vaccine [16]. Harberts et al. showed a 94% immune response assessed among 36 LT recipients, including seroconversion among 60% (3/5) of non-responders after the third vaccine [17].

This study aimed to prospectively assess the safety and efficacy of a fourth dose of the BNT162b2 vaccine against the Omicron variants of concern among LT recipients and to compare the rate of breakthrough SARS-CoV-2 infections among LT recipients vaccinated with four versus three doses of the vaccine.

## 2. Materials and Methods

**Figure 1:**
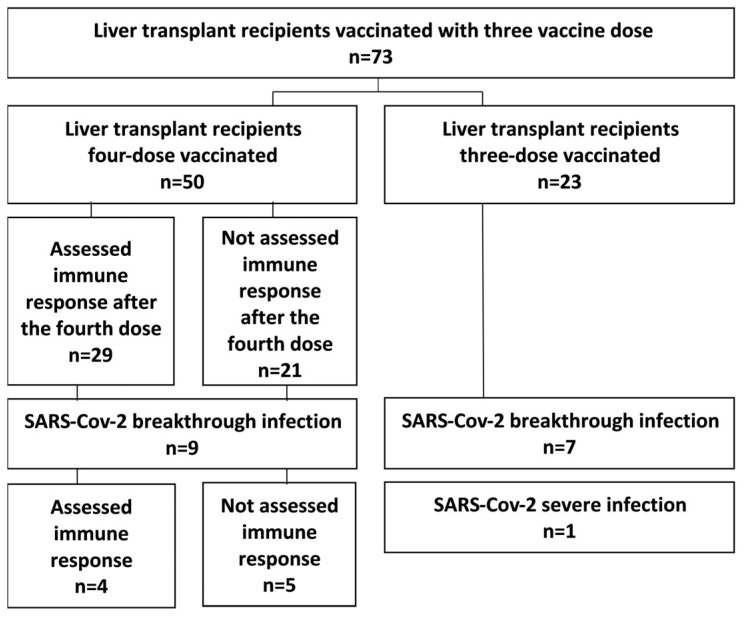
Flowchart of the study cohort.

This study was conducted at the Sheba Medical Center between 21 December 2021 and 1 May 2022 and was approved by the institutional review board (8008-20-SMC). All participants agreed to participate in the study and signed informed consent before any study-related procedures were conducted. The study population included 73 adults (age > 18 years old). LT recipients were routinely followed at the Liver Diseases Center. LT recipients who received three doses of the BNT162b2 vaccine, who had no past or active SARS-CoV-2 infection, and who had an assessment of immune response after the third vaccine were eligible to participate in the study. The flowchart of the entire cohort according to vaccination status is presented in Figure 1.

Out of the 73 LT recipients vaccinated with three doses of the BNT162b2 vaccine, 50 were vaccinated with the fourth vaccine. The immune response after the fourth vaccine was assessed in 29 of 50 patients.

Demographic, clinical, and laboratory data were extracted from electronic patient records. Blood tacrolimus or everolimus trough levels were determined, and routine blood tests were performed between the time the fourth vaccine was administered and before the serology test in patients who received the fourth vaccine and during the follow-up period in patients who did not. Renal function was calculated using the chronic kidney disease epidemiology collaboration (CKD-EPI) creatinine equation. Chronic kidney disease was defined as eGFR < 60 mL/min/1.73 m^2^ for a duration of >3 months.

### 2.1. Serology Assay

#### 2.1.1. Antibody Detection Testing

Blood samples were centrifuged at 4000× *g* for 4 min at room temperature. Serum titers of IgG antibodies against the SARS-CoV-2 spike RBD were quantified using the commercial automatic chemiluminescence microparticle immunoassay SARS-CoV-2 IgG II Quant (Abbott, Chicago, IL, USA), according to the manufacturer’s instructions. IgG antibody titers > 21.4 international binding antibody units per milliliter (BAU/mL) were defined as positive (responders), while anti-RBD IgG < 21.4 BAU/mL was defined as negative (non-responders). A SARS-CoV-2 pseudo-virus (psSARS-2) neutralization assay was performed, as previously described [17], to detect SARS-CoV-2 neutralizing antibodies. The assay used a green fluorescent protein (GFP) reporter-based pseudotyped virus with a vesicular stomatitis virus (VSV) backbone coated with the SARS-CoV-2 spike (S) protein that was generously provided by Dr. Gert Zimmer (Institute of Virology and Immunology (IVI), Mittelhäusern, Switzerland). Sera not capable of reducing viral replication by 50% at 1 to 8 dilutions or below were considered non-neutralizing.

#### 2.1.2. Viral Isolation of the Wild-Type and Omicron Variants and SARS-CoV-2 Micro-neutralization Assays

Wild-type and VOC were isolated from nasopharyngeal samples from SARS-CoV-2-positive individuals which contained the wild-type sublineage B.1.1.50 (hCoV-19/Israel/CVL-45526-ngs/2020), B.1.1.529, Omicron, and BA.1 (hCoV-19/Israel/CVL-n49814/2021) variants. Confluent VERO-E6 cells were incubated for 1 h at 33 °C with 300 μL of nasopharyngeal samples, after which 5 mL 2% FCS 3MEM-EAGLE medium was added. Upon CPE detection, supernatants were aliquoted and stored at −80 °C, as previously described [18]. VERO-E6 cells were seeded in sterile 96-well plates with 10% FCS MEM-EAGLE medium and incubated at 37 °C for 24 h. One hundred TCID50 of wild-type and Omicron (BA.1 and BA.2) SARS-CoV-2 isolates were incubated with inactivated sera diluted from 1:8 to 1:16, 384 in 96-well plates for 60 min at 33 °C. Virus-serum mixtures were placed over the VERO-E6 cells and incubated for 5 days at 33 °C, after which gentian violet stain (1%) was applied to fix and stain the cell culture layer. The neutralizing dilution of each serum sample was determined by identifying the well with the highest serum dilution without an observable cytopathic effect. A dilution of 1:10 or above was considered neutralizing [19].

### 2.2. Statistical Analysis

Continuous variables are presented as median (IQR) for skewed distributions. Categorical variables are expressed as count (percentage). Antibody titers were log-transformed before the statistical analysis. Titers of anti-RBD SARS-CoV-2 IgG and of neutralizing antibodies (NA) were calculated for all groups and are presented as geometric mean titers (GMT) with a 95% confidence interval (CI). Categorical variables were compared using a chi-squared analysis and Fisher’s exact test. Continuous measurements were compared by Student’s *t*-test or the Mann–Whitney U test, according to their distribution. Non-parametric Wilcoxon paired tests were conducted to compare quantitative data. To assess predictors of maintenance of immune response after the third dose, patients were categorized by their anti-RBD IgG titer. Correlations between anti-RBD IgG and NA titers were estimated using the Spearman correlation test. *p* < 0.05 was considered a statistically significant difference. All tests were two-sided. Statistical analysis was performed with SPSS (IBM SPSS Statistics, version 25, IBM Corp., Armonk, NY, USA, 2016). Scatter plots of the analyzed data were produced using GraphPad Prism version 9.2.0 for Windows (GraphPad Software, San Diego, CA, USA).

## 3. Results

### 3.1. Baseline Characteristics

Baseline demographics and clinical and laboratory characteristics of the 73 LT recipients are summarized in Table 1.

The median patient age was 63.8 years (interquartile range (IQR) 52.1–70.4 years; range: 26–83 years); 51% were male. Median time since the LT was 9.2 years (IQR 5.3–16.4 years). Six patients (8.2%) had undergone combined liver and kidney transplantation. Comorbidities were frequent, with hypertension (53.4%), diabetes mellitus (41.1%), chronic kidney disease (38.4%), and dyslipidemia (46.6%) being the most common. Calcineurin inhibitors (CNI) were the principal immunosuppressive agent administered to seventy-one patients (sixty-five tacrolimus and six cyclosporine). CNI monotherapy was administered to thirty-eight patients (53.5%), while twenty-eight patients (38.4%) were receiving double immunosuppression therapy and six (8.2%) patients were receiving triple (Table 2).

Serum samples were collected from LT recipients at a median of 132 days (interquartile range [IQR]), 130–138 days) after the third vaccine and 29 days (IQR, 25–33) after the fourth dose. The median time between the third vaccine follow-up serology assessment and administration of the fourth vaccine dose was 38 days (IQR 29–44).

Four months after the third vaccine, a positive humoral immune response was detected in 56 of the 73 LT recipients (77.8%). Responders to the third vaccine after one- and four-month follow-ups were lower among patients who did not receive the fourth vaccine dose compared to those who received it (75% vs. 83.3%, *p* = 0.5, and 69.6% vs. 81.6%, *p* = 0.3, respectively) (Table 1).

### 3.2. Humoral Immunity to the Fourth BNT162b2 mRNA Vaccine Dose

Fifty LT recipients were vaccinated with the fourth vaccine. Baseline demographics, clinical, and laboratory characteristics of the 50 LT recipients vaccinated with the fourth dose are presented in Table 1. The immune response after the fourth vaccine was assessed in 29 of 50 patients. Paired levels anti-RBD IgG, psSARS-2 NA (ps-NA), and NA against wild-type and Omicron BA.1 were available for 25 of 29 patients who had participated in our previous study [6], enabling comparison of the humoral immune response shortly after the third vaccine (first test) (n = 25, at a median time of 22 days (IQR, 22–28 days)), at a later stage after the third vaccine dose (second test) (n = 29, at a median time of 131 days (IQR, 129–136 days)), and after the fourth vaccine dose (third test) (n = 29, at a median time of 29 days (IQR, 25–33 days)). The median time between the third and the fourth vaccine doses was 175 days (IQR, 164–176 days).

**Figure 2:**
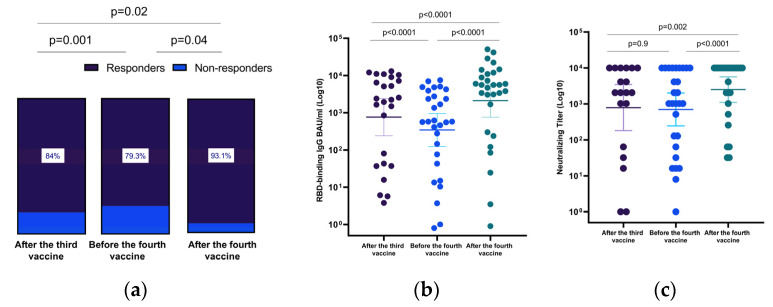
Changes in the humoral immune response after the third and the fourth vaccine doses among liver transplant recipients. (**a**) Changes in immune response after the third and fourth doses among the 29 liver transplant recipients who had paired serology samples. (**b**) Scatter plot presenting changes in anti-RBD IgG and (**c**) SARS-CoV-2 pseudo-virus neutralizing antibody titers after the third and the fourth vaccine dose (after the third vaccine test, serum collection was 22 days (IQR, 21–28 days), after the third vaccine and before the fourth vaccine test, it was 131 days (IQR, 129–137 days), and after the third dose and after the fourth vaccine test, it was 29 days (IQR, 25–33 days) after administration of the fourth vaccine dose). Differences in paired samples were calculated using the Wilcoxon matched-pairs signed-rank test. The horizontal line indicates geometric mean values with a 95% confidence interval.

The humoral immune responses of the 29 patients with paired serology samples were compared and presented in Figure 2 and Table 3.

Four months after the third vaccine (before the fourth vaccine), the immune response was detected in 23/29 (79.3%) of patients (Table 3, Figure 2). Geometric mean anti-RBD IgG decreased significantly four months after the third vaccine as compared to the levels measured one month after the third vaccine (766 BAU/mL (95% CI, 241–2428) to 345 BAU/mL (95% CI, 124–955), *p* < 0.0001; geometric mean ps-NA levels decreased from 785 (95% CI, 180–3434) to 699 (95% CI, 244–2008); however, these differences were not significant (*p* = 0.9) (Figure 2, Table 3). Geometric mean anti-RBD IgG and ps-NA levels increased significantly after the fourth dose as compared to levels measured before its administration (345 BAU/mL (95% CI, 124–956) to 2118 BAU/mL (95% CI, 761–5900) *p* < 0.0001 and 699 (95% CI, 244–2008) to 2489 (95% CI, 1098–5640), *p* < 0.0001, respectively (Figure 2).

Due to the small sample size and the highly significant response to the fourth dose among the LT recipients, predictors of negative immune response after the fourth dose could not be evaluated (Table 4).

Immediately after the fourth booster, the immune response was enhanced significantly, from 84% to 93.1% (*p* = 0.02), with geometric mean anti-RBD IgG and ps-NA levels significantly higher after the fourth dose as compared immediately after the third vaccine (766 BAU/mL (95% CI, 241–2428) to 2118 BAU/mL (95% CI, 761–5900), *p* = 0.001, and 785 (95% CI, 180–3434) to 2489 (95% CI, 1098–5640), *p* = 0.002, respectively) (Figure 2).

Titers of ps-NA correlated positively with anti-RBD IgG titers (r = 0.81, *p* < 0.0001; r = 0.84, *p* < 0.0001; r = 0.85, *p* < 0.0001 after the third vaccine, before and after fourth doses, respectively).

Titers of ps-NA after the fourth dose correlated negatively with combined immunosuppression (double and triple therapy) compared to CNI monotherapy (r = −0.5, *p* = 0.004). No correlation was found between ps-NA titers following the fourth dose and age, sex, comorbidities (diabetes mellitus, hypertension, dyslipidemia, and impaired renal function), or subtypes of immunosuppression treatment (MMF, mTOR, or prednisone).

### 3.3. Neutralization of Wild-Type and Omicron Variants (BA.1 and BA.2 Variants)

**Figure 3:**
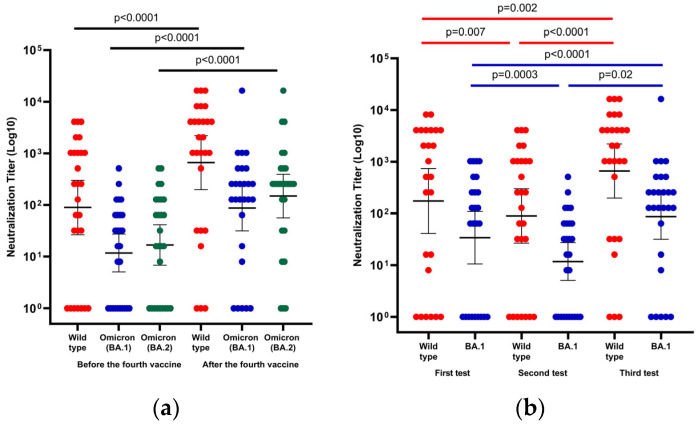
Neutralization efficiency against the wild-type SARS-CoV-2 virus and Omicron variants of concern BA.1 and BA.2. (**a**) Scatter plot presenting changes in wild-type, BA.1 and BA.2 Omicron neutralizing antibodies before and after the fourth vaccine; (**b**) Changes in titers of neutralizing antibody against the wild-type virus and BA.1 variant among 27 liver transplant recipients who had paired serology samples after the third and the fourth vaccine dose (in the first test, serum collection was 22 days (IQR, 21–28 days) after the third vaccine, in the second test it was 131 days (IQR, 129–137 days) after the third dose, and in the third test it was 29 days (IQR, 25–33 days) after administration of the fourth vaccine dose). Differences in paired samples were calculated using the Wilcoxon matched-pairs signed-rank test. The black horizontal line indicates geometric mean values with a 95% confidence interval.

The efficacy of neutralizing antibodies against wild-type and Omicron variants BA.1 and BA.2 was tested before and after the fourth vaccine. The GMT of wild-type and Omicron variants BA.1 and BA.2 was assessed before the administration of the fourth vaccine in 71 of 73 patients and was 79 (95% CI, 39–159), 10 (95% CI, 6–15), and 15 (95% CI, 9–25) (*p* < 0.0001). The GMT of wild-type and Omicron variants BA.1 and BA.2 after the fourth vaccine was assessed in 27 of 29 patients and was 662 (95% CI, 198–2209), 87 (95% CI, 32–240), 149 (95% CI, 57–394), *p* < 0.0001 and *p* = 0.001, respectively (Figure 3).

The NA to BA.1 titer after the fourth dose correlated positively with anti-RBD IgG titers (r = 0.7, *p* < 0.0001). No correlation was found between NA BA1 titers following the fourth dose and age, sex, comorbidities (diabetes mellitus, hypertension, dyslipidemia, and impaired renal function), or subtypes of immunosuppression treatment.

The NA to BA2 titers after the fourth dose correlated positively with anti-RBD IgG titers (r = 0.82, *p* < 0.0001) and negatively with combined immunosuppression (double and triple therapy) compared to CNI monotherapy (r = −0.403, *p* = 0.04). No correlation was found between NA BA2 titers following the fourth dose and age, sex, comorbidities (diabetes mellitus, hypertension, dyslipidemia, and impaired renal function), or subtypes of immunosuppression treatment (MMF, mTOR, or prednisone).

Next, we performed a comparison in paired samples neutralizing efficacy against wild-type and Omicron variants BA.1 at three timepoints (immediately after the third vaccine, before the fourth vaccine, and after the fourth vaccine) in twenty-seven patients. The GMT of wild-type BA.1 immediately after the third vaccine (first test), that was 174 (95% CI, 41–737) and 34 (95% CI, 11–109), decreased significantly in the second test (before the fourth vaccine) and improved significantly after the fourth vaccine (third test); 89 (95% CI, 27–301), 12 (95% CI, 5–27) and 662 (95% CI, 198–2209), 87 (95% CI, 32–241), respectively. It is important to mention that the level of neutralizing antibodies against the BA.1 variant improved significantly after the fourth vaccine compared to their level immediately after the third vaccine (*p* < 0.0001) (Figure 3).

### 3.4. Breakthrough Infection

The entire cohort was analyzed for the rate of breakthrough infections. No difference in clinical and laboratory characteristics was found between LT patients who received three vs. four vaccine doses (Table 1). A breakthrough infection was documented in sixteen patients, with nine (18%) contracting a SARS-CoV-2 infection after the fourth vaccine dose and seven (30.4%) after the third dose (*p* = 0.2). The median time between SARS-CoV-2 infection and the fourth dose was 49 days (IQR, 29–60 days; range, 21–111 days). Breakthrough infections were mild in 15/16 (93.5%) cases, while one patient who had received three vaccine doses developed a severe infection and required hospitalization. No fatal cases were documented (Table 5).

Breakthrough infections after the fourth vaccine positively correlated with the presence of diabetes mellitus (r = 0.4, *p* = 0.03), showing a higher prevalence among diabetic (7/21 (33.3%)) as compared to nondiabetic (2/29 (6.9%)) recipients (*p* = 0.02). Age, gender, other comorbidities, type of immunosuppression therapy, anti-RBD, and ps-NA levels after the fourth vaccine dose did not correlate with breakthrough infections.

**Figure 4:**
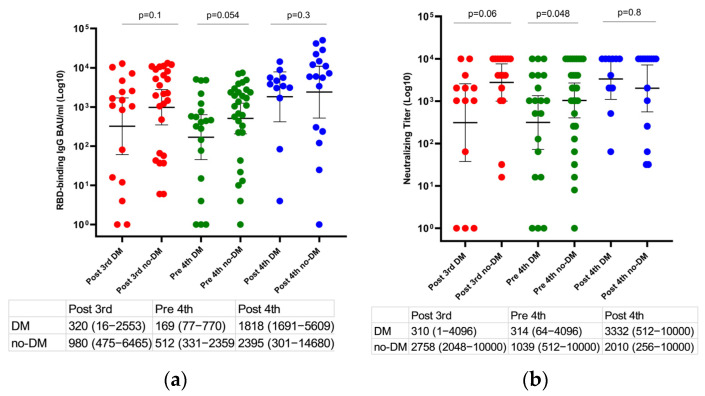
Changes in the humoral immune response after the third and the fourth vaccine doses among liver transplant recipients with and without diabetes mellitus. (**a**) Scatter plot presenting changes in anti-RBD IgG and (**b**) SARS-CoV−2 pseudo-virus neutralizing antibody titers after the third and the fourth vaccine dose (post-third serum collection was 22 days (IQR, 21–28 days) after the third vaccine, the pre-fourth was 131 days (IQR, 129–137 days) after the third dose, and the post-fourth was 29 days (IQR, 25–33 days) after administration of the fourth vaccine dose). Differences in paired samples were calculated using the Wilcoxon matched-pairs signed-rank test. The horizontal line indicates geometric mean values with a 95% confidence interval.

The GMT anti-RBD IgG and ps-NA were compared in our LT recipients’ group with and without diabetes mellitus. The GMT anti-RBD IgG and ps-NA were numerically higher in most time points among LT recipients without diabetes mellitus compared to the diabetes group. Only the titer of ps-NA was significantly higher among non-diabetic LT recipients compared to diabetics (314 (64–4096) vs. 1039 (512–10000), *p* = 0.048 (Figure 4). However, the GMT anti-RBD IgG and ps-NA titers were not different between patients with and without breakthrough infections divided by diabetes mellitus.

**Figure 5:**
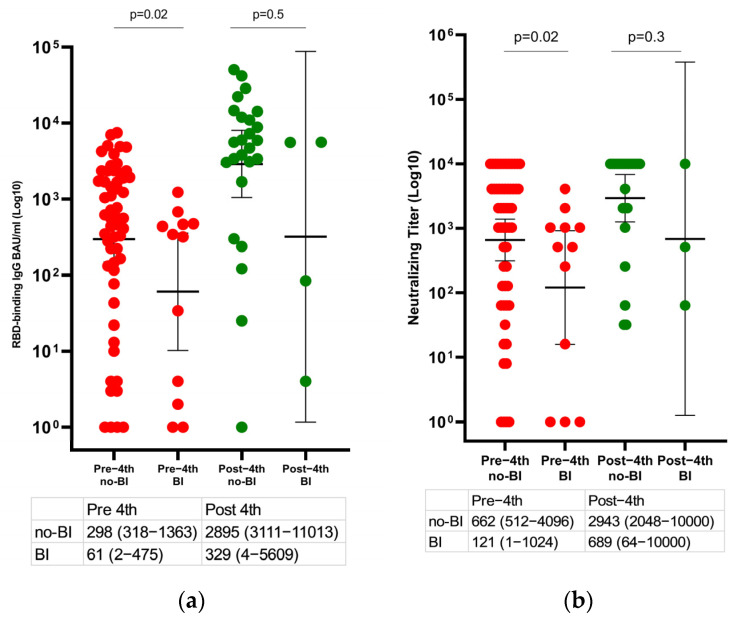
Changes in the humoral immune response after the third and the fourth vaccine doses among liver transplant recipients with and without a breakthrough infection. (**a**) Scatter plot presenting changes in anti-RBD IgG and (**b**) SARS-CoV−2 pseudo-virus neutralizing antibody titers before and after the fourth vaccine dose (the pre-fourth was 131 days (IQR, 129–137 days) after the third dose and the post-fourth was 29 days (IQR, 25–33 days) after administration of the fourth vaccine dose). Differences in paired samples were calculated using the Wilcoxon matched-pairs signed-rank test. The horizontal line indicates geometric mean values with a 95% confidence interval. BI, breakthrough infection.

The GMT anti-RBD IgG and ps-NA were compared in our LT recipients’ group with and without breakthrough infection (Figure 5).

The GMT anti-RBD IgG and ps-NA before the fourth vaccine was significantly higher among recipients without than with a breakthrough infection and was 2895 BAU/mL (95% CI, 3111–11013) vs. 61 BAU/mL (95% CI, 2–475), *p* = 0.02; 662 (95% CI, 512–4096) vs. 121 (95% CI, 1–1024), *p* = 0.02, respectively. The GMT anti-RBD IgG and ps-NA after the fourth dose was numerically lower among LT recipients with a breakthrough infection than without. However, the differences were not statistically significant.

### 3.5. Adverse Effects of the Fourth BNT162b2 mRNA Vaccine Dose

Adverse effects (mostly mild) that were reported by 25% of patients included shoulder pain, muscle pain, headache, and fatigue.

## 4. Discussion

We aimed to assess the humoral immune response of LT recipients to the fourth BNT162b2 mRNA vaccine and also the immune response to the currently widespread VOC. The fourth dose of the vaccine significantly improved humoral immune responses among LT recipients. The positive immune response reached 84% one month after the third vaccine dose, waned to 79.3% within the subsequent three months, and significantly improved to 93.1% one month after the fourth vaccine dose. Our findings align with a recent report of improved immune response with the fourth vaccine dose among immunocompetent patients [7,9].

The vaccine effectiveness against VOC was assessed before and after the fourth vaccine. The neutralizing antibody titer against the Omicron variant BA.1 and BA.2 was significantly lower than the wild-type of SARS-CoV-2 virus before and after the fourth vaccine. Moreover, the neutralizing antibody titers against the BA.2 Omicron variant were significantly higher than the BA.1 Omicron variant.

Moreover, our important findings demonstrated that the immune response after the fourth vaccine was significantly higher than the immune response immediately after the third boost at all levels (anti-RBD IGG, ps-NA, and NA titers of BA.1). We have detected a difference between the changes in levels of anti-RBD IgG and ps-NA immediately after the third vaccine and three months after the third vaccine. Although the titer of anti-RBD IgG decreased significantly, the levels of ps-NA did not change significantly. However, we have demonstrated, a positive correlation in both anti-RBD IgG and ps-NA titers at one and four months after the third vaccine (manuscript submitted). The difference in titers is most likely due to the significant increase in the strength of interaction between IgG antibodies and SARS-CoV-2 antigen (avidity) observed after the third dose. These findings are in line with a recent publication that has demonstrated that the third vaccine dose among HCWs elicits IgG affinity maturation, which specifically affects neutralization capacity [20]. This finding suggests that lower IgG antibody levels will be required to maintain high neutralizing titers and, as a result, immunity may be sustained for a longer period following vaccination [20].

The recent emergence of the Omicron variant affected the efficacy of the currently available COVID-19 vaccines and the administration of neutralizing antibody therapeutics [21]. The immune response of twenty-four immunocompetent patients after the three doses of the BNT162b2 mRNA vaccine showed significantly lower BA.2 and BA.1 neutralizing antibody titers than the wild-type [13]. Additionally, a clinical observational study during the Omicron wave showed that an additional boost among nursing home residents provided higher protection than the two doses [21]. Data on the effectiveness of the fourth vaccine dose among LT recipients are limited. The improved immune response after the fourth as compared to the third vaccine dose was associated with fewer numerical incidences of breakthrough infections. However, the difference did not reach statistical significance, likely due to the small sample size. In a recent follow-up of immunocompetent HCWs at our center, breakthrough infection incidence was shown to correlate with antibody titers [13]. Bar-On et al. also reported that the fourth dose provided added short-term protection against confirmed SARS-CoV-2 infections, with incidence twofold and the rate of severe disease threefold lower four weeks after vaccination in comparison to eligible individuals who did not receive the fourth dose [7]. Peled et al. reported an improved immune response among HT recipients after the fourth BNT162b2 vaccine dose, from 61.4% before to 80.7% after the fourth dose [15]. Midtvedt et al. reported a 42% positive immune response to the fourth dose among non-responder kidney transplant (KT) recipients [16].

In line with our previously reported observation [1,6], the present study found a significant association between the immune response and the type of immunosuppression therapy (combined immunosuppression vs. CNI monotherapy). Recently, a dose-dependent effect of MMF on the immune response has been reported among KTRs; KTRs taking < 1 g MMF per day had a better immune response to mRNA vaccines than patients on higher dosage regimens (OR 5.19, 95% CI 1.49–18.00, *p* = 0.009) [22].

It was previously reported that advanced age, metabolic risk factors, and immunocompromised conditions impaired immune response to the BNT162b2 vaccine [23,24,25]. In a recent review, Norbert Stefan [23] stressed that hyperglycemia and insulin resistance might drive immunosenescence and concluded that these risk factors might also promote vaccine-breakthrough SARS-CoV-2 infections in fully vaccinated individuals. Indeed, we found that breakthrough infection incidence after the fourth vaccine was significantly higher among diabetic patients.

One of the strengths of the present study lies in its pioneering attempt to evaluate immune responses and breakthrough infection rates among LT recipients who received the fourth vaccine dose. We excluded the LT recipients who were SARS-CoV-2 infected at different time points of the COVID-19 pandemic in order to have a homogenous uninfected LT recipients group. Using this strategy allowed us to draw a more accurate conclusion regarding the effectiveness of the vaccine. The study was limited by its small sample size. In the present study, a trend of avoidance of vaccination with the fourth dose was noted among LT recipients with an earlier negative immune response, which may have influenced our cohort’s high response rate to the fourth vaccine. Thus, a definitive conclusion regarding the protection against severe infection provided by the fourth vaccine dose cannot be made at the present time. Additionally, this study did not present an assessment of T-cell response.

## 5. Conclusions

In summary, the fourth BNT162b2 mRNA vaccine significantly improved humoral immune responses among LT recipients. The fourth vaccine dose was not associated with severe adverse effects. NA titers after the fourth dose correlated negatively with combined immunosuppression compared to CNI monotherapy. The breakthrough infection incidence was lower than among recipients who received three doses, but the difference did not reach statistical significance. The rate of breakthrough infection was higher among diabetic patients. Further studies with a larger sample size are needed to evaluate additional factors potentially influencing the breakthrough infection rate among LT recipients receiving the fourth vaccine dose.

## Figures and Tables

**Table 1:** Baseline demographics, clinical, and laboratory characteristics of patients who received three vs. four doses of the BNT162b2 mRNA vaccine.

Characteristics	Total LT Cohort n = 73	Three Vaccine Doses n = 23	Four Vaccine Doses n = 50	*p*
Age, years	63.8 (52.1–70.4)	65.3 (44.7–70.1)	62.7 (53.1–70.6)	0.8
Male, n (%)	37 (51)	11 (47.8)	26 (52)	0.7
Indication for LT				0.3
Hepatitis C, n (%)	16 (21.9)	5 (21.7)	11 (22)	
NASH, n (%)	16 (21.9)	2 (8.7)	14 (28)	
Hepatitis B, n (%)	6 (8.2)	4 (17.4)	2 (4)	
PSC, n (%)	9 (12.3)	1 (4.3)	8 (16)	
PBC, n (%)	3 (4.1)	1 (4.3)	2 (4)	
ALD, n (%)	5 (6.8)	3 (13)	2 (4)	
Other, n (%) ^¶^	18 (24.7)	7 (30.4)	11 (22)	
Age at transplantation, years	52.5 (41.9–62.1)	49 (30.8–60.3)	53 (44.8–62.8)	0.2
Time since liver transplantation, years	9.2 (5.3–16.4)	12.4 (5.3–23.3)	7.7 (5.3–15.5)	0.3
Comorbidities				
Diabetes mellitus, n (%)	30 (41.1)	9 (39.1)	21 (42)	0.8
Hypertension, n (%)	39 (53.4)	12 (52.2)	27 (54)	0.8
Dyslipidemia, n (%)	34 (46.6)	11 (47.8)	23 (46)	0.8
Chronic kidney disease, n (%)	28 (38.4)	10 (43.5)	18 (36)	0.5
BMI, kg/m^2^	25.8 (22.3–27.9)	24.7 (21–27.9)	26.2 (22.7–27.9)	0.5
WBC, K/microL	5.7 (4.7–6.8)	7 (5.1–7.2)	5.4 (4.5–6.7)	0.2
Hemoglobin, g/dL	13 (11.8–14.3)	13.3 (10.9–14)	13 (12–14.7)	0.1
Platelets, K/microL	168 (131–217)	170 (121–191)	164 (131–221)	0.6
Creatinine, mg/dL	1 (0.81–1.25)	1.0 (0.8–1.3)	1.0 (0.8–1.2)	0.9
ALT, IU/L	20.5 (15–29.5)	23 (14–34)	20 (16–27)	0.9
ALP, IU/L	100 (75–134)	109 (84–130)	99.5 (74.5–137.5)	0.6
Bilirubin, mg/dL	0.6 (0.5–0.8)	0.6 (0.4–0.9)	0.6 (0.5–0.8)	0.9

^¶^ Other indications for liver transplantation: biliary atresia, cystic fibrosis, fulminant liver failure, Budd–Chiari syndrome, sarcoidosis, Wilson disease, autoimmune hepatitis, secondary sclerosing cholangitis, and glycogen storage disease. ALT, alanine aminotransferase; ALP, alkaline phosphatase; BMI, body mass index; CNI, calcineurin inhibitor; IQR, interquartile range; LT, liver transplantation; MMF, mycophenolate mofetil; NASH, non-alcoholic steatohepatitis; PBC, primary biliary cholangitis; PSC, primary sclerosing cholangitis.

**Table 2:** Type of immunosuppression treatment and immune response of patients who received three vs. four doses of the BNT162b2 mRNA vaccine.

Characteristics	Total LT Cohort n = 73	Three Vaccine Doses n = 23	Four Vaccine Doses n = 50	*p*
Tacrolimus dose, mg/trough level, ng/mL	3 (2–4)/5 (4–7)	3 (2–4)/5 (4–7)	3 (2–4)/5 (4–6)	0.8/0.8
Prednisone, n (%)/dose, mg	13 (17.8)/5 (5–8)	4 (17.4)	9 (18)	0.9/0.3
MMF, n (%)/dose mg	21 (28.8)/100 (720–1000)	6 (26.1)/1000 (720–1000)	15 (30)/1000 (500–1000)	0.7/0.9
Everolimus, n (%)/dose mg/trough level, ng/mL	10 (13.7)/2 (2–3)/4 (3–6)	1 (4.3)/1/4	9 (18)/2 (2–3)/4 (3–6)	0.1/0.2/1
CNI monotherapy, n (%)	38 (53.5)	13 (56.5)	25 (52.1)	0.7
Double ^‡^/triple, n (%) ^‡‡^ immunosuppression	28 (38.4)/6 (8.2)	10 (43.5)/0	18 (36)/6 (12)	0.2
SARS-CoV-2 infection	16 (21.9)	7 (30.4)	9 (18)	0.2
Positive immune response * one month after the third vaccine ^§^	47 (81)	12 (75)	35 (83.3)	0.5
Positive immune response * four months after the third vaccine ^§§^	56 (77.8)	16 (69.6)	40 (81.6)	0.3

^‡^ Double immunosuppression denotes the combination of CNI and MMF—thirteen patients; CNI and everolimus—eight patients; CNI and prednisone—seven patients. ^‡‡^ Triple immunosuppression therapy was administered to six patients (the combination of CNI, MMF, and prednisone—five patients; the combination of mTOR inhibitors, MMF, and prednisone—one patient). * A positive immune response was defined as IgG antibody titers > 21.4 BAU/mL. ^§^ The immune response was assessed at a median time of 22 days (IQR, 21–28 days) after the third vaccine dose. ^§§^ The immune response was assessed at a median time of 132 days (IQR, 130–138 days) after the third vaccine dose.

**Table 3:** Immune response before and after the fourth BNT162b2 mRNA vaccine compared to immune response immediately after the third vaccine.

Characteristics	First Test One Month after the Third Dose ^‡^ n = 25	Second Test Four Months after the Third Dose ^‡‡^ n = 29	Third test One Month after the Fourth Dose ^‡‡‡^ n = 29
Positive anti-RBD IgG *, n (%)	21 (84)	23 (79.3)	27 (93.1)
50% SARS-CoV-2 pseudo-virus neutralization titer, GM (CI 95%)	785 (180–3434)	699 (244–2008)	2489 (1098–5640)
Anti-RBD IgG titer, BAU/mL, GM (CI 95%)	766 (241–2428)	345 (124–955)	2118 (761–5900)
50% wild-type neutralization titer, GM (CI 95%)	174 (41–737)	89 (27–301)	662 (198–2209)
50% Omicron BA.1 neutralization titer, GM (CI 95%)	34 (11–109)	12 (5–27)	87 (32–241)

^‡^ Serum was collected at a median time of 22 days (IQR, 21–28) after administration of the third vaccine dose. ^‡‡^ Serum was collected at a median of 131 (IQR, 129–137) days after administration of the third vaccine dose. The wild-type and Omicron BA.1 neutralization titers were assessed in 27 of 29 patients. ^‡‡‡^ Serum was collected at a median of 29 days (IQR, 25–33 days) days after administration of the fourth vaccine dose. The wild-type and Omicron BA.1 neutralization titers were assessed in 27 of 29 patients. * A positive anti-RBD IgG was defined as IgG antibody titers > 21.4 BAU/mL.

**Table 4:** The Humoral immune response of 29 patients after being vaccinated with a fourth dose of the BNT162b2 mRNA vaccine.

Characteristics	Serology Assessment after the Fourth Vaccine n = 29	Non-Responders n = 2	Responders n = 27	*p*
Age, years	64.2 (54.3–70.4)	64.8 (54.3–75.4)	64 (49.2–10.4)	0.8
Male, n (%)	16 (55.2)	1 (50)	15 (55.6)	0.7
Age at transplantation, years	54.2 (41–63.1)	59.4 (53.4–65.4)	54.2 (40.1–63.1)	0.5
Time since liver transplantation, years	7.5 (4.3–15)	5.4 (0.9–9.97)	7.5 (4.3–15.2)	0.4
Comorbidities				
Diabetes mellitus, n (%)	12 (41.4)	1 (50)	11 (40.7)	0.7
Hypertension, n (%)	13 (44.8)	1 (50)	12 (44.4)	0.7
Dyslipidemia, n (%)	12 (41.4)	1 (50)	11 (40.7)	0.7
Chronic kidney disease, n (%)	10 (34.5)	1 (50)	9 (33.3)	0.6
BMI, kg/m^2^	25.3 (22.1–27)	20.9 (15.8–26.1)	25.3 (22.1–27.4)	0.4
WBC, K/microL	5.7 (4.8–6.6)	4.5 (4.2–4.7)	5.7 (4.8–6.7)	0.1
Hemoglobin, g/dL	13.1 (12.4–15.5)	11.1 (10.2–12)	13.6 (12.4–16)	0.04
Creatinine, mg/dL	0.97 (0.8–1.2)	1.2 (1.1–1.3)	0.9 (0.8–1.2)	0.2
ALT, IU/L	19 (17–26)	20.5 (17–24)	19 (16–27)	1.0
Prednisone, n (%)	3 (10.3)	0	3 (11.1)	0.8
MMF, n (%)	8 (27.6)	1 (50)	7 (25.9)	0.5
Everolimus, n (%)	4 (13.8)	1 (50)	3 (11.1)	0.3
CNI monotherapy, n (%)	13 (44.8)	0	16 (59.3)	0.1
Double/triple immunosuppression, n (%)	11 (37.9)/2 (6.9)	2 (100)	9 (33.3)/2 (7.4)	0.2
SARS-CoV-2 infection, n (%)	3 (10.3)	0	3 (11.1)	0.8

Patients with IgG antibody titers > 21.4 BAU/mL were defined as responders. Patients with titers < 21.4 BAU/mL were defined as non-responders. ALT, alanine aminotransferase; BMI, body mass index; CNI, calcineurin inhibitor; IQR, interquartile range; LT, liver transplantation; MMF, mycophenolate mofetil.

**Table 5:** Comparison of baseline characteristics of 50 patients who received a fourth BNT162b2 mRNA dose vaccine with and without breakthrough infections.

Characteristics	No SARS-CoV-2 Infection n = 41	SARS-CoV-2 Infection n = 9	*p*-Value
Age, years	61.8 (54–71)	66.4 (52–70.4)	0.8
Male, n (%)	20 (48.8)	6 (66.7)	0.9
Age at transplantation, years	53 (45–60)	54 (45–63)	0.9
Comorbidities			
Diabetes mellitus, n (%)	14 (34.1)	7 (77.8)	0.016
Hypertension, n (%)	21 (51)	6 (66.7)	0.4
Dyslipidemia, n (%)	18 (44)	5 (55.6)	0.5
Chronic kidney disease, n (%)	14 (34)	4 (44.4)	0.5
BMI, kg/m^2^	25 (22–28)	27 (26–28)	
WBC, K/microL	5.7 (4.7–7.0)	4.6 (3.9–5.2)	0.05
Hemoglobin, g/dL	13 (12.3–15.2)	13.7 (11.5–14.1)	0.6
Creatinine, mg/dL	0.97 (0.8–1.2)	1.23 (1.0–1.5)	0.096
ALT, IU/L	19 (16–26)	26.5 (15.5–44.5)	0.4
Prednisone, n (%)	8 (19)/5 (5–6)	1 (11.1)/5	0.5/1
MMF, n (%)	11 (26.8)/1000 (750–1500)	9 (100)/625 (500–875)	0.3/0.2
Everolimus, n (%)	4 (3–6)/9 (22)	0	0.1/NA
CNI monotherapy, n (%)	20 (51.3)	5 (55.6)	0.8
Double/triple immunosuppression, n (%)	14 (34.1)/6 (14.6)	4(44.4)/0	0.6

## Data Availability

Not applicable.
